# Beyond the current state of just-in-time adaptive interventions in mental health: a qualitative systematic review

**DOI:** 10.3389/fdgth.2025.1460167

**Published:** 2025-01-28

**Authors:** Claire R. van Genugten, Melissa S. Y. Thong, Wouter van Ballegooijen, Annet M. Kleiboer, Donna Spruijt-Metz, Arnout C. Smit, Mirjam A. G. Sprangers, Yannik Terhorst, Heleen Riper

**Affiliations:** ^1^ Clinical, Neuro-, and Developmental Psychology, Vrije Universiteit Amsterdam, Amsterdam, Netherlands; ^2^ Amsterdam Public Health, Mental Health, Amsterdam, Netherlands; ^3^ Department of Medical Psychology, Amsterdam UMC Location University of Amsterdam, Amsterdam, Netherlands; ^4^ Unit of Cancer Survivorship, German Cancer Research Center (DKFZ), Heidelberg, Germany; ^5^ Department of Psychiatry, Amsterdam UMC, Amsterdam, Netherlands; ^6^ Center for Economic and Social Research, University of California, Los Angeles, CA, United States (emeritus); ^7^ Department of Psychiatry, Interdisciplinary Center Psychopathology and Emotion Regulation, University Medical Center Groningen, University of Groningen, Groningen, Netherlands; ^8^ Department of Psychology, Ludwig Maximilian University of Munich, Munich, Germany; ^9^ German Center for Mental Health (DZPG), Partner Site Munich-Augsburg, Munich, Germany

**Keywords:** just-in-time adaptive intervention, digital mental health, intervention development, smartphone intervention, JITAI

## Abstract

**Background:**

Just-In-Time Adaptive Interventions (JITAIs) are interventions designed to deliver timely tailored support by adjusting to changes in users' internal states and external contexts. To accomplish this, JITAIs often apply complex analytic techniques, such as machine learning or Bayesian algorithms to real- or near-time data acquired from smartphones and other sensors. Given the idiosyncratic, dynamic, and context dependent nature of mental health symptoms, JITAIs hold promise for mental health. However, the development of JITAIs is still in the early stages and is complex due to the multifactorial nature of JITAIs. Considering this complexity, Nahum-Shani et al. developed a conceptual framework for developing and testing JITAIs for health-related problems. This review evaluates the current state of JITAIs in the field of mental health including their alignment with Nahum-Shani et al.'s framework.

**Methods:**

Nine databases were systematically searched in August 2023. Protocol or empirical studies self-identifying their intervention as a “JITAI” targeting mental health were included in the qualitative synthesis if they were published in peer-reviewed journals and written in English.

**Results:**

Of the 1,419 records initially screened, 9 papers reporting on 5 JITAIs were included (sample size range: 5 to an expected 264). Two JITAIs were for bulimia nervosa, one for depression, one for insomnia, and one for maternal prenatal stress. Although most core components of Nahum-Shani's et al.'s framework were incorporated in the JITAIs, essential elements (e.g., adaptivity and receptivity) within the core components were missing and the core components were only partly substantiated by empirical evidence (e.g., interventions were supported, but the decision rules and points were not). Complex analytical techniques such as data from passive monitoring of individuals' states and contexts were hardly used. Regarding the current state of studies, initial findings on usability, feasibility, and effectiveness appear positive.

**Conclusions:**

JITAIs for mental health are still in their early stages of development, with opportunities for improvement in both development and testing. For future development, it is recommended that developers utilize complex analytical techniques that can handle real-or near-time data such as machine learning, passive monitoring, and conduct further research into empirical-based decision rules and points for optimization in terms of enhanced effectiveness and user-engagement.

## 1 Introduction

Over the past two decades, a variety of digital interventions have been developed, evaluated, and implemented for the prevention and treatment of a range of mental health disorders, including depression, anxiety and substance use disorders (1–7). The diverse range of digital interventions include standalone guided and unguided, as well as blended (i.e., integration of digital and face-to-face components into one treatment protocol) or add-on formats, with treatments often based on cognitive behavioral therapy (CBT) (1–7). Digital interventions have demonstrated potential in improving clients' self-management skills, enhancing access to psychological interventions, and expanding mental health care at relatively lower costs (1, 2, 4) while achieving comparable clinical outcomes compared to traditional face-to-face interventions [e.g., (2, 7–10)]. While digital interventions were initially concentrated on internet-based platforms, in the past decade, the development, large ownership, and intensive use of smartphone functionalities have made it possible to deliver interventions via smartphones (11–14). Smartphone interventions enhance monitoring capabilities and enable real-time prevention and treatment options (4). Reviews indicate that smartphone interventions are potentially acceptable and effective across various mental health conditions, such as depression and anxiety (14–19).

A novel form of smartphone intervention is the Just-In-Time Adaptive Intervention (JITAI). JITAIs automatically offer support and dynamically adjust their type, content, timing, and intensity based on an individual's changing status and setting, and are ideally only initiated when needed (20, 21). Measuring individual's changing status and setting is often conducted using “ecological momentary assessment” (EMA) methods (22–27). EMA captures momentary symptoms, emotions, behaviors, and context, providing insights that are difficult to achieve through retrospective methods (22–27). They can be used for clinical and epidemiological research as well as in clinical practices, and EMA data can be collected through both active and passive methods (22, 24). In active EMA, users self-monitor their symptoms and related factors (e.g., social contacts and context) by completing short questionnaires, typically several times a day depending on the mood/behavior under study (22–24). Passive EMA refers to monitoring without active engagement of the user, such as counting the number of steps or geolocations, using deployable sensors or sensors in smartphones or smartwatches (22, 28). This data can inform decision-making about when, if, and which intervention should be offered (29). In making these decisions, various complex analytical techniques such as machine learning can be employed to classify the individual's state and context (20, 21, 30). These can range from simple if-then rules and decision trees over more complex supervised algorithms, such as support vector machines (SVM) to end-to-end learning models and large multimodal models (20, 21, 29–34). To date, investigations into JITAIs have primarily focused on addressing health challenges such as physical inactivity (35), obesity (36), smoking (37), and substance abuse (38). For example, HeartSteps, a JITAI designed to promote physical activity (39, 40). When passive EMA and a machine learning algorithm detect sedentary behavior, HeartSteps sends push notifications with interventions aimed at increasing physical activity. The machine learning algorithm determines the appropriate intervention based on various conditions. For instance, on weekends when individuals are at home and the weather is good, a suggested activity might be walking 500–1,000 steps. While during weekday work hours or bad weather conditions, a suggested activity might be standing up (39, 40). Meta-analytic findings demonstrate moderate to large effect sizes of JITAIs on distal outcomes such as weight loss and smoking cessation when compared to no-treatment control conditions and non-JITAI treatments (41).

JITAIs for mental health are still in their early stages. However, they are perceived as a promising future direction in the field of mental health (12, 13, 42). One reason is that a substantial body of research indicates that mental health symptoms are idiosyncratic (i.e., differ between individuals), dynamic (i.e., fluctuate over time), and multi-factorial (i.e., driven by multiple variables, such as setting, time of day, and personal characteristics) (26, 43–46). As a result, different individuals with mental health problems require personalized types of support in real-life situations, and the same individual may also need different forms of support at different moments in time (11, 47). JITAIs seem suitable for intervening in mental health symptoms, given that these interventions are based on individual characteristics and changing contexts and states within individuals.

Developing JITAIs, however, is a difficult task since JITAIs are considered complex interventions due to the multifactorial nature of (mental) health-related problems and intervention components (20, 21). Many decisions, ideally theory- and/or empirically-based, must be made during development about when to intervene, when to abstain from intervention, which interventions should be delivered to whom, and in which specific settings these modules can best be delivered. These choices include designing the right interventions, determining the factors on which to base these decisions, and deciding which methods will be used to measure and analyze the outcomes (20, 21). To support JITAI development, Nahum-Shani et al. (20, 21) introduced a conceptual framework designed to aid JITAI development for health-related problems by systematically considering core components and guide subsequent empirical work. This framework comprises six core components. (1) Distal outcome represents the long-term goal of the intervention, typically the primary clinical outcome. (2) Proximal outcome refers to short-term goals the intervention aims to achieve, often serving as mediators through which the interventions impact the distal outcome. (3) Intervention options refer to the array of possible interventions that can be employed to achieve the proximal outcome. (4) Tailoring variables pertain to information used to determine when and how to intervene for each individual. This includes “vulnerability/opportunity” and “receptivity”. Vulnerability refers to experiencing adverse health outcomes or engaging in maladaptive behaviors. For example, in a JITAI addressing depression, perhaps the best time to provide an intervention is when an increase in depressed mood is detected. However, on occasions where a vulnerable state may not be ideal for intervening (e.g., during stress), it may be better for a JITAI to adapt to states of “opportunity”. Receptivity pertains to the user's availability to receive and engage with the provided intervention effectively. For instance, for a JITAI for depression, this might be the time when the user is at home, as engaging with the intervention requires time and space. Often, information about vulnerability and receptivity is gathered through EMA methods and then analyzed using machine learning or other types of algorithms in JITAIs. Ideally, the algorithms used have the capacity to “learn” when a person is most receptive to a particular intervention. (5) Decision points are points in time at which an intervention may be delivered. (6) Decision rules, which are rules specifying which intervention to offer, to whom, and when. These rules systematically link decision points, tailoring variables, and intervention options. Nahum-Shani et al. emphasize that the six core components need to be substantiated based on behavioral change theory (20, 21).

The goal of this systematic review is to investigate the current state of JITAIs for mental health, and to assess the extent to which the identified JITAIs for mental health align with the Nahum-Shani et al. framework (20, 21). The secondary objective is to describe the studies on JITAIs for mental health in terms of their characteristics, aims, and outcomes. Furthermore, the review also aims to identify potential knowledge gaps. Through this, insights to guide the future development of JITAIs for mental health are drawn.

## 2 Methods

### 2.1 Overview, eligibility criteria, information sources and search

The protocol was registered with the Open Science Framework on 23 February 2023 (https://osf.io/4u27c/c). The review follows the updated PRISMA 2020 guidelines (48).

Studies were included if (1) the focus was on JITAIs interventions, only studies with interventions that were self-identified as “JITAIs” were included, (2) the intervention was targeted at (subclinical) mental disorders, (3) it concerned a protocol or empirical study, (4) it was published in a peer-reviewed journal, (5) written in English.

To identify relevant articles, a specialist librarian searched the following nine databases from inception until 01 Aug 2023: PubMed, Embase, APA PsycInfo, Cinahl, Web of Science, Cochrane CENTRAL, ACM Digital Library, DBLP, and Scopus. The search string is shown in Supplementary Table 1. The reference lists of relevant studies were checked for potentially eligible studies by two reviewers (AS, CvG). In PubMed, the following query was used: “JITAI” [tiab] OR “JITAIs” [tiab] OR “just-in-time adaptive intervention*” [tiab] OR [“just-in-time” (tiab) AND adaptive (tiab)] OR “momentary intervention*” [tiab]. To keep the terms as broad as possible and ensure no studies were missed due to overly strict criteria, no terms related to mental health were added. For the other databases, similar queries were used (see “Supplementary Table 1 Search Queries”).

### 2.2 Study selection

To identify papers that potentially met the eligibility criteria, titles and abstracts were screened independently by two reviewers (MT, CvG). The full text of relevant studies identified after the title and abstract screening were reviewed by two reviewers (AS or MT, CvG) to assess inclusion eligibility. Disagreements on study selection were resolved by mutual consensus or during discussion with the project principal investigator (HR), if needed.

### 2.3 Data extraction and synthesis

After inclusion of eligible studies, relevant data was extracted using data charting forms. First, general information was extracted, that is authors, publication year, country, and target population. Then, for the primary objective, which is the extent to which the identified JITAIs align with Nahum-Shani et al.'s framework (20, 21), the operationalization of core JITAI components based on Nahum-Shani et al.'s framework (20, 21) (i.e., distal outcome, proximal outcome, intervention options, tailoring variables, decision points, decision rules) was extracted. Subsequently, for the secondary aim, which is to describe the studies in terms of their characteristics and outcomes, information about the study aim, target population, study design, control group, sample size, recruitment and inclusion criteria, primary outcome, and main findings was extracted.

Two researchers (AS or MT, CvG) independently extracted and charted the data, discussed the results of the included studies, and updated the data charting forms in an iterative process. Disagreements between the two researchers were resolved by discussion with the project principal investigator (HR), if needed.

## 3 Results

### 3.1 Study selection and general study characteristics

**Figure 1:**
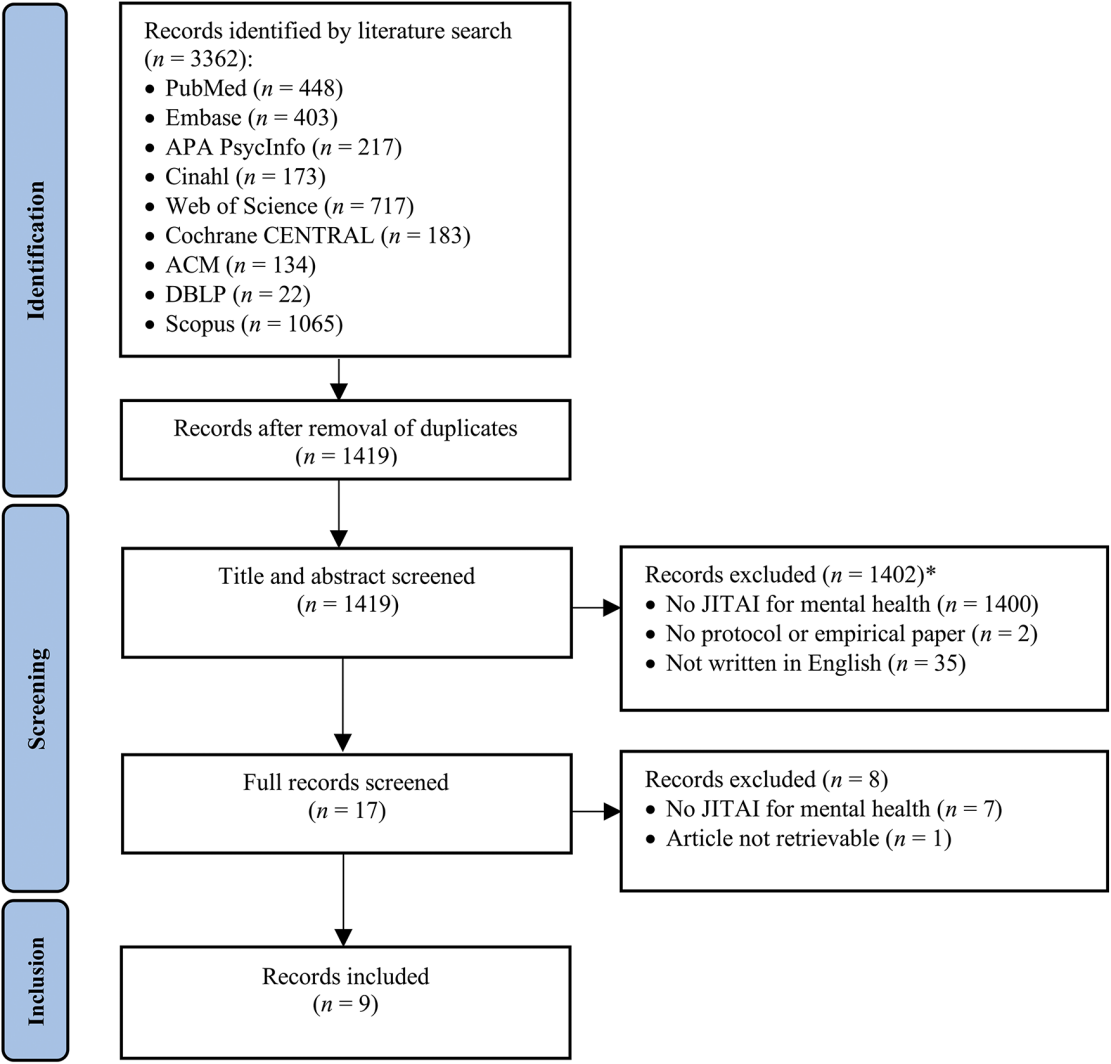
PRISMA flowchart of the study selection process. *35 papers neither had a focus on JITAI nor were written in English.

The search resulted in 3,362 articles, 1,419 after duplicate removal. The flowchart of the inclusion process following PRISMA guidelines is presented in Figure 1. 1,402 articles were excluded based on title and abstract screening. Of the remaining 17 articles, 8 were excluded after full text screening, leaving 9 articles for inclusion. The studies were published between 2018 and 2023.

Five papers targeted Bulimia Nervosa Spectrum Disorders (BNSD) (49–53). Four of these discussed the “CBT+” JITAI intervention (49–52), while the fifth described the “SenseSupport” JITAI (53). Both JITAIs were delivered alongside standard weekly CBT face-to-face sessions. One paper focused on depression and introduced a 3-week stand-alone mobile rumination-focused CBT known as “JITAI-MRFCBT” to address depressive rumination (54). Two papers focused on insomnia and described the guided “iREST” JITAI, aimed at addressing clinical sleep disturbances in military personnel (55, 56). Finally, one paper focused on maternal prenatal stress and introduced “Wellness-4-2”, a JITAI delivered alongside a validated 12-session face-to-face prenatal maternal stress reduction course (57).

### 3.2 JITAI development: operationalization of JITAIs

The operationalization of the JITAIs according to the core components of Nahum-Shani et al.'s framework (20, 21) is described in Table 1. For a detailed description per JITAI, please refer to “Supplementary Material 1 JITAI development: Operationalization of JITAIs”. Below is a summary at an aggregated level. In total, five distinct JITAIs were described in nine papers.

**Table 1:** Operationalization of the JITAI components.

JITAI (disorder)	Distal outcome	Proximal outcome	Intervention options	Tailoring variables	Decision points	Decision rules
CBT+ (BNSD)	Increasing the utilization and acquisition of six core CBT skills that help patients reach the main clinical outcome (e.g., reduce dietary restraints such as regular eating and increase adaptive responses to cues such as manage binge eating).	• Practice with core CBT skills	Different interventions are available for each specific CBT skill, interventions are messages. All interventions (1) provide a brief rationale why the patient would benefit from practicing that CBT skill, (2) provide guidance on how to implement the suggested skill at the current time.	• **Variable 1:** Patient set three weekly goals (e.g., regular eating) in collaboration with their clinician. These weekly goals are typically linked to one of the six CBT skills.	Each time a patient completes an EMA. Patients are encouraged to open the app themselves and receive a notification if a certain amount of time has elapsed since their last meal entry (based on their individual eating schedules).	The algorithm (decision tree) checks the CBT skills in the three weekly goals (**variable 1**), checks whether the EMAs suggest an at-risk state linked to this weekly goal (**variable 2, 3, 4 & 5**), checks the conditions set by the clinician (**variable 6**).
		• Reach weekly priority goals (the goals are typically linked to one of the six CBT skills)				
				• **Variable 2:** Patients record all eating episodes (e.g., food consumed).		
				**• Variable 3:** Patients report disordered eating behavior (e.g., laxative use).		
				• **Variable 4:** Patients report strong urge to engage in disorder eating behavior.		
				• **Variable 5:** Patients report when they experienced a significant change in mood.		
				• **Variable 6:** Clinicians can choose interventions that may be relevant for their patients and could scale up the frequency of the notifications.		
SenseSupport (BNSD)	Reduce dietary restraint.	Reduce dietary restrictions.	Interventions are in-the-moment reminders augmenting the therapeutic content of the regular CBT sessions (e.g., when the algorithm detects fasting behavior, the system delivers an intervention encouraging the patient to eat regularly throughout the day).	• **Variable:** Glucose levels (Dexcom G6 continuous and passive glucose monitoring sensor).	The glucose monitoring sensor has readings every five minutes.	The parameter invariant-algorithm analyses the (changes in) glucose levels to detect meal consumption and more specifically, ED behavior: including binge eating, purging, and fasting behavior (**variable**).
JITAI-MRFCBT (Depression)	Reduction of depressive rumination.	Disruption ruminative thoughts.	Interventions are CBT-based messages and training exercises tailored to the type of trigger. Content can include necessary problem-solving skills, motivation, and support.	**• Variable 1:** EMAs of stressful events in the past three hours.	Each time a patient completes an EMA (five times a day, three-hour interval)	The algorithm (decision tree) checks if the person experienced a stressful event in the past three hours (**variable 1**) and checks whether this potential event was a trigger for rumination (**variable 2**) and whether the person is receptive (**variable 4**). If conditions are not met, **no intervention**. In case conditions are met, intervention is sent with content of intervention matched to the nature of the trigger (**variable 3**).
				**• Variable 2:** In the case of a stressful event in the past three hours (**variable 1**), EMA to assess whether it is a trigger for rumination.		
				**• Variable 3:** The nature of the trigger (**variable 2**) assessed with EMA.		
				**• Variable 4:** Daily activity survey to assess receptivity in the form of not engaging in other activities (e.g., driving and walking).		
iREST (Insomnia)	Reduction of insomnia.	Not specified.	Personalized CGT based sleep tips (e.g., sleep restriction) on how to address and overcome certain behaviors, cognitions, or events (e.g., nightmares) that may be perceived as barriers to healthy sleep.	• **Variable 1:** Wake logs (i.e., daytime activities that may impact healthy sleep).	Each time a patient completes an EMA. That is, wake logs in the evening and sleep logs in the morning.	The algorithm (decision tree) suggests appropriate sleep tips based on the wake (**variable 1),** sleep logs (**variable 2**) and the routine outcome measures (**variable 3**). Then the clinician makes judgments if the intervention suggested by the algorithm is fitted (**variable 4**). Clinicians can also self-initiate certain interventions.
				• **Variable 2:** Sleep logs (e.g., sleep quality).		
				• **Variable 3:** Weekly routine outcome measures (e.g., sleep, PTSD).		
				• **Variable 4:** Clinicians makes judgment which intervention to deliver.		
Wellness-4-2 (Maternal prenatal stress)	Improve fetal neurodevelopment.	Decrease maternal prenatal perceived stress.	Interventions are CBT based text messages focused on skill reinforcement and mindfulness.	• **Variable 1:** Heart rate (measured via unobtrusive wireless ECG sensor).	Not specified.	The algorithm (decision tree) checks elevated stress detection, based on heart rate (**variable 1**) and/or subjective stress reports (**variable 2**) and matches content to the most recent f-t-f sessions (**variable 3**).
				• **Variable 2:** 4 EMAs of subjective stress a day.		
				• **Variable 3:** Content from the most recent f-t-f intervention session.		

BNSD, bulimia nervosa spectrum disorder; CBT, cognitive behavioral therapy; ECG, electrocardiogram; EMA, ecological momentary assessment.

#### 3.2.1 Distal outcomes

Concerning distal outcomes, Nahum-Shani et al.'s framework (20, 21) was adhered to across all JITAIs (49–57). The outcomes represented clinical outcomes, mostly derived from the treatment outcome of face-to-face CBT, specific for the mental health disorder targeted. For instance, within the target population of military personnel experiencing clinically significant sleep disturbances, insomnia served as the distal outcome (55, 56).

#### 3.2.2 Proximal outcomes

For the four JITAIs that specified the proximal outcomes (49–54, 57), the selection of these outcomes largely adhered to Nahum-Shani et al.'s (20, 21). The proximal outcomes were theoretically justified based on non-JITAI (mainly face-to-face CBT) interventions, with the treatment mechanisms of these non-JITAI interventions considered as the JITAI's proximal outcomes. For instance, in face-to-face CBT for depressive rumination, treatment is focused on disrupting ruminative thoughts in the short term since this leads to a reduction in these thoughts over the long term (58). In the JITAI-MRFCBT, disrupting ruminative thoughts in the short term was the proximal outcome (54). Only CBT+'s proximal outcome was adaptive between and within users, with the user's proximal outcome changing weekly (49–52). Adaptivity was not reported in the other JITAIs (53, 54, 57).

#### 3.2.3 Intervention options

Most interventions adhered to this aspect of Nahum-Shani et al.'s framework (20, 21). Each intervention was primarily based on face-to-face CBT with an emphasis on behavioral change. Different interventions were sent to and among participants based on tailored variables (49–57). Four out of the five JITAIs linked the positive impact of interventions to a positive impact on the proximal outcome (49, 51–54, 57). The exception was iREST, which linked their interventions to a positive impact on the distal outcome (55, 56). The interventions in all the five JITAIs were adaptive as they were adjusted both between and within users over time (49–57).

#### 3.2.4 Tailoring variables

For the operationalization of the tailoring variables, some aspects of Nahum-Shani et al.'s framework (20, 21) were adhered to, but essential elements within the component were missing. Diverse methods were utilized for (self)monitoring, with active EMA in four JITAIs (49–52, 54–57), passive EMA in two (53, 57) sometimes supplemented with other sources such as weekly retrospective monitoring or clinician involvement (49, 51, 55, 56). Most choices for the tailoring variables were based on face-to-face CBT. However, the tailoring variables were not adaptive between and within users over time. Moreover, only JITAI-MFRCBT assessed both states of vulnerability and receptivity (54). In the other four JITAIs, only vulnerability was considered (49–53, 55).

#### 3.2.5 Decision points

The decision points were specified in four out of five JITAIs and Nahum-Shani et al.'s framework (20, 21) was partly followed (49–56). SenseSupport's decision point occurred every five minutes, so the likelihood of missing a user's moment of need for an intervention was low (53). However, in the other JITAIs, there was a longer interval between decision points (49–52, 54–56). In two JITAIs, the decision points occurred after the active EMAs were completed (49–52, 54), and with iREST, adjustments were made only after the clinician had approved the intervention (55, 56). The decision points were not adaptive between and within users over time. Since this frequency was not based on theory, it cannot be determined whether the timing of interventions (and thus the “in-time” concept) is accurate or if crucial moments were missed (20, 21).

#### 3.2.6 Decision rules

Regarding the decision rules, Nahum-Shani et al.'s framework (20, 21) was partially followed. All JITAIs employed machine learning algorithms for decision rules (49–57). Four used decision trees of varying complexity (49–52, 54–57), while SenseSupport utilized a more complex algorithm (i.e., parameter invariant-algorithm) (53). CBT+ and iREST included online clinician involvement (49–52, 55, 56), which is not described in Nahum-Shani et al.'s framework (20, 21). As with most other components, decision rules were not adaptive between and within users over time (49–57).

### 3.3 Studies on JITAIs in mental health research

The general characteristics, aim of the study, target population, study design, control group, sample size, recruitment and inclusion criteria, primary outcome, and main findings of the nine conducted studies are described in Table 2. For detailed narrative descriptions per study, please refer to “Supplementary Material 2 Narrative descriptions of the included studies”. Below is a summary at an aggregated level.

**Table 2:** Characteristics of the included studies.

Author, year, country	Study aim	Target population	Study design (control group)	Sample size	Recruitment and inclusion criteria	Primary outcome	Main findings
Bulimia Nervosa Spectrum Disorder (CBT+)							
Juarascio et al., 2021, USA	Describe the development of CBT+ JITAI and present the feasibility, acceptability, and preliminary outcome data from a small proof-of-concept pilot trial for BN.	Individuals with BN	Pilot study (no control)	*n* = 5 patients*, n* *=* 3 clinicians	• Recruitment not specified • Primary diagnosis BN; age 18–70; no active severe psychiatric comorbidity; no intellectual disability; ability to speak and write English	• Feasibility: subjective appraisal app use and user adherence to self-monitoring (EMA) • Acceptability: qualitative patient and clinician feedback • Preliminary outcomes: improvements in CBT skills utilization and acquisition, reduction in BN symptoms	• Feasibility: patients reported low burden and adherence to the EMAs was high (M = 3.13, SD = 1.03, per day). Though study dropout was relatively high (i.e., 40%) • Acceptability: CBT+ was perceived as useful by both patients and clinicians • Preliminary outcomes: Large improvements in CBT skills and clinically significant improvement in BN symptoms were observed post-treatment
Juarascio et al., 2023a, USA	Assess the feasibility and acceptability of the CBT+ JITAI in conjunction with weekly face-to-face CBT sessions, evaluate the ability of JITAIs to improve skill utilization, examine pre- to post-treatment changes in BN symptoms in individuals receiving CBT+ JITAIs and provide a preliminary estimate of the efficacy of JITAIs to inform the design of a future fully powered RCT.	Individuals with BN	RCT in which all participants received weekly CBT sessions (control group used the app for self-monitoring, but did not receive a JITAI)	*n* = 55 (*n* = 29 experimental group, *n* = 26 control group)	• Recruited through flyers, radio and social media • >11 binge episodes & >11 compensatory behaviors in the last 3 months; age 18–70; BMI >17.5; no medical complications that prohibit safe outpatient treatment; no comorbid diagnoses such as psychotic disorder; no developmental disorder; no current or planned pregnancy; no history of bariatric surgery	• Feasibility: in-app retention rate • Acceptability: statements based on the Technology Acceptance Model • Target engagement: BNSD skill use • Treatment outcomes: BN symptoms (EDE)	• Feasibility: JITAIs demonstrated feasible (89.7% retention at post-treatment). • Acceptability: Participants rated the JITAI as acceptable • Target engagement: Both groups demonstrated greater BNSD skill use for dietary restraint, regular eating, incorporating feared foods, adaptive responses to cues and urge management (within-group *p*'s < 0.05), with no difference between groups (between group *p*'s > 0.05) • Treatment outcomes: Both groups showed significant improvements in BN symptoms (within-group *p*'s < 0.5), with no difference between groups (between-group *p*'s > 0.05).
Presseller et al., 2022, USA	Examine whether emotion regulation deficits and impulsive behavior at pre-treatment are moderators for pre-to-post change in BN symptoms by treatment condition (CBT+ JITAI vs. no-JITAI)	Individuals with BN	Secondary analyses	See Juarascio et al. 2023a (51)	See Juarascio et al. 2023a (51)	Past month frequency of BN symptoms and global eating pathology, measured with the EDE 17.0	• Higher emotion regulation deficits predict greater pre-to-post treatment improvement in compensatory behaviors in the JITAI condition compared to the control group • Elevated impulsivities predict greater pre-to-post treatment improvement in compensatory behavior and binge eating in the JITAI condition compared to the control group.
Juarascio et al., 2023b, USA	To describe the protocol of a full factorial RCT that will evaluate the optimal complexity level of two commonly used mHealth components (self-monitoring and micro interventions) alongside CBT and will test if this optimal complexity level of interventions is moderated by baseline self-regulation.	Individuals with BN or BED	2 × 3 full factorial RCT (All participants receive 16 weekly CBT sessions and are randomized to one of the six treatment conditions determined by a combination of 1) self-monitoring (standard self-monitoring and skills monitoring OR standard self-monitoring) and 2) micro intervention condition (CBT+ JITAI OR automated reminder messages OR no micro intervention)	Expected *n* = 264 (*n* *=* 44 per condition)	• Recruited from the community • DSM-5 criteria BN or BED; age 18–65; no BMI <18.5; no current ED treatment; no medical complications that prohibit safe outpatient treatment; no severe psychopathology	• Optimal intervention: evaluate the optimal intervention based on past month frequency of BN symptoms and global eating pathology measured with the EDE 17.0 at the end of treatment and 6-, and 12- month follow-up. • Moderation: evaluate whether the optimal intervention is moderated by baseline deficits in self-regulation	NA
Bulimia Nervosa Spectrum Disorder (SenseSupport)							
Juarascio et al., 2022, USA	To describe the feasibility, acceptability, target engagement, and initial treatment outcome of SenseSupport when used in conjunction with 12 weekly sessions of CBT.	Individuals with BN or BED	ABAB design. In which SenseSupport JITAI is turned on (A) for 2 weeks and then turned off for two weeks (B) throughout a 12-week treatment period next to weekly sessions of outpatient CBT (participants act as their own control group).	*n* = 30	• Recruited through professional referrals and radio, newspaper and web-based (social media) advertisements • DSM-5 diagnosis BN or BED; no current treatment for ED or behavioral weight loss; at least 18 years; not requiring immediate treatment for medical complications because of the ED; no severe psychopathology; unstable on psychiatric medications for at least 1 month; no diabetes; no medication known to impact insulin or glucose levels; no history of bariatric surgery; no current pregnancy or lactating; no BMI <17.5 or >40	• Feasibility: Retention in the study and percentage of data obtained from the continuous glucose monitoring sensors • Acceptability: qualitative participant ratings in questionnaires (e.g., the comfort, ease of use, helpfulness of the JITAI) and interviews • Target engagement (weekly measured): EDE-Q restraint subscale (overall dietary restraint, avoidance of eating for ≥ 8 h; desire for an empty stomach, food avoidance, and dietary rules); dietary restraints (frequency of fasting ≥5 h over the past 7 day) • Treatment outcomes: EDE global score; number of binge eating episodes and compensatory behaviors in the previous 7 days.	• Feasibility: Retention in the study was high (25/30, 83% after treatment), but rates of continuous glucose monitoring data collection were low (67.4% of expected data was collected). • Acceptability: Participants reported that the SenseSupport system was comfortable, minimally disruptive, and easy to use. • Target engagement: The desire for an empty stomach was statistically significantly lower in the JITAI-On weeks than in the JITAI-Off weeks (Between Cohen's *d* = 0.25). No other statistically significant differences. • Treatment outcomes: Participants demonstrated statistically significant large decreases in binge eating (within Cohen's *d* = 2.07), compensatory behaviors (within Cohen's *d* = 0.68), and global eating pathology (within Cohen's *d* = 1.25) from pre-to-posttreatment
Depression (JITAI-MRFCBT)							
Wang & Miller, 2023, USA	Describe the results of the pilot RCT on a JITAI for mobile rumination-focused CBT (JITAI-MRFCBT) for depressive rumination compared to a no-treatment control condition.	Adults in therapy for MDD	2-armed pilot RCT (no-treatment control group)	*n* = 18 (*n* = 9 experimental group, *n* = 9 control group)	• Recruited via the volunteering website “ResearchMatch”, a web-based registry for new, potentially disorder-relevant studies. • Age 18 years or older; self-reported having been given a diagnosis of clinical depression in the past 12 months; self-reported that they had no diagnosis of any other mental health related disorder; ability to speak, read and write English; smartphone with a data plan	Reduction in count of rumination episodes and average duration of each ruminative episode from baseline to week 5 (post-intervention for experimental condition). Assessed with 5 EMAs a day, 7 days baseline and 7 days post-intervention.	• Post-intervention participants in the experimental group, compared with those in the control group, reported greater reduction in counts of rumination episodes (M = −25.28, SD = 14.50 vs. M = 1.44, SD = 4.12, *p* < .001, Between Cohen *d* = 2.5) and greater reduced average time (minutes) spent in rumination (M = −21.53, SD = 17.6, vs. M = 1.47, SD = 1.5, *p* = .04, between Cohen *d* = 1.84).
Insomnia (iREST)							
Pulantara et al., 2018a, USA	Develop and assess the usability of the iREST JITAI for delivering evidence-based sleep interventions and explore the potential effectiveness of this treatment delivery form relative to in-person delivery.	Active-duty service members and veterans (age 18–60 years) with clinically significant sleep disturbances	• Description of development • Pilot usability study (no control group)	*n* = 19	• Recruited through postcards, flyers, study website, social media, and public television. • Significant sleep complaints for at least 1 month; no history of psychotic or bipolar disorders; no sleep apnea (current or past); no severe or untreated psychiatric disorder; not pregnant or lactating; no scheduled/imminent military deployment during the study.	System usability (SUS, TUQ, quantitative feedback)	Post treatment participants (17/19) rated the app as highly useable (SUS of 85.76, SD = 12.37), were satisfied with the app and would consider using it in the future (TUQ = 4.31/5, SD = 0.63).
Pulantara et al., 2018b, USA	Evaluate the effectiveness of the iREST JITAI for delivering evidence-based sleep interventions and explore the potential effectiveness of this treatment delivery form relative to in-person delivery.	Active-duty service members and veterans (age 18–60 years) with clinically significant sleep disturbances	Assessment of clinically significant decrease in insomnia severity, and non-inferiority test compared to care as usual (no control group)	*n* = 27	• Recruited from other studies that used postcards, flyers, study websites, social media/Facebook (San Francisco, CA), and public television advertisements for recruiting purposes • Significant sleep disturbances (ISI >9), for at least one month; no history of psychotic or bipolar disorders; no sleep apnea (current or past); no narcolepsy; no severe or untreated psychiatric disorder; not pregnant or lactating.	Insomnia (ISI), sleep quality (PSQI)	• Significant pre to post intervention improvements in insomnia severity [mean reduction on the ISI of 9.96, t(26) = 9.99, *p* < .001]. • Post treatment, 70% (19/27) of participants met the criteria for treatment response and 59% (16/27) achieved remission. • These response and remission rates showed no significant differences with previously published results for in-person trials.
Maternal prenatal stress (Wellness-4-2)							
Wakschlag et al., 2021, USA	Describe the development of and the protocol of an RCT for the Wellness-4-2 JITAI for women with prenatal stress.	Women with prenatal stress	RCT (control group receives TAU for pregnant women)	Anticipated *n* = 100 (*n* = 50 experimental group, *n* = 50 control group)	Second trimester of pregnancy	Infant trajectories of dysregulation (brain and behavioral markers) across the first year of life	NA

BED, binge eating disorder; BN, bulimia nervosa; BNSD, bulimia nervosa spectrum disorders, CBT, cognitive behavioral therapy; EDE, eating disorder examination interview; EDE-Q, eating disorder examination questionnaire (self-report), EMA, ecological momentary assessment; ISI, insomnia severity index; JITAI, just-in-time adaptive intervention; NA, not applicable, PSQI, Pittsburgh sleep quality index; RCT, randomized controlled trial; RRS, ruminative response scale; SUS, system usability score; TAU, treatment as usual, TUQ, telerehabilitation usability questionnaire.

Four papers reported on the usability and/or feasibility, with most findings revealing that users perceived the JITAIs as feasible and usable (49, 51, 53, 55). Feasibility was evaluated either by study retention rates or app usage data. Three of the four papers assessed the usability of the JITAI with rating scales that were based on the Technology Acceptance Model, and one study used the System Usability Scale and a modified Telerehabilitation Usability Questionnaire. In addition, qualitative interviews or feedback were used in all studies to assess usability. Five papers reported a change in distal outcomes from pre-to-pos*t*-test in small pilot (RCT) studies, showing within-person decreased symptomatology from pre-to-post intervention (49, 51, 54–56). In three papers, comparisons were made between the JITAI groups and control groups (51, 54, 56), of which two were RCTs and in one paper the control group consisted of data extracted from previously published trials on face-to-face CBT for insomnia. These studies found that JITAIs led to a greater reduction in symptoms when compared to a non-active control group (54), but no difference in reduction in symptoms was found when compared to an active control group (51, 56). In two studies (38, 40), attention was also given to subgroups, examining whether JITAIs (e.g., offering a JITAI vs. not offering a JITAI) moderated treatment outcomes for specific subgroups based on BNSD symptom profiles (50, 52). Last, two papers were protocols of RCTs, that started recruiting in 2023.

## 4 Discussion

This review identified nine papers describing a total of five distinct JITAIs for mental health, targeting BSND, insomnia, depressive rumination, and maternal prenatal stress. Regarding the first aim, three out of the five JITAIs described all six core components of Nahum-Shani et al.'s framework (20, 21), while two JITAIs lacked one core component (i.e., a proximal outcome or decision point). However, not all essential elements within the core components were incorporated (e.g., receptivity and adaptivity as part of the tailoring variables) and there is uncertainty regarding whether the interventions were delivered “in-time” or whether crucial moments were missed since the decision points were not theory-based but based on pragmatic choices (e.g., being able to ask users to complete a few EMAs a day). No components outside Nahum-Shani et al.'s framework (20, 21) were identified. Regarding the second aim, two papers were protocols of RCTs' that started recruiting in 2023. The other studies demonstrated that the JITAIs were generally usable, feasible, and led to a reduction in mental health symptoms compared to pre-intervention. In studies with a control group, the reduction was greater than in no-treatment control conditions but did not significantly differ from non-JITAI interventions.

### 4.1 Gaps of knowledge and recommendations for developing and testing JITAIs for mental health

Based on the comparison of the alignment of JITAIs in mental health with Nahum-Shani et al.'s framework (20, 21) and the status of the conducted studies, we identified gaps of knowledge and make several recommendations for future development and testing of JITAIs in mental health. This will aid in optimizing personalization, effectiveness, and user engagement in JITAIs (20, 21).

#### 4.1.1 The use of passive EMA and (complex) machine learning techniques

Receptivity was only included in JITAI-MRFCBT, assessed through a daily questionnaire where users self-reported their expected windows of receptivity (43). JITAIs in other domains sometimes measure receptivity using passive EMA. For example, in the HeartSteps JITAI for physical activity and sedentary behavior, phone-based accelerometer data is analyzed by machine learning algorithms at each decision point to determine the user's receptivity by determining the user's current activity (39, 40). A benefit of using passive EMA to measure receptivity compared to active EMA is that it does not rely on users completing questionnaires, reducing user burden and minimizing the risk of missing data (20, 21, 59). Moreover, passive EMA has been shown to incrementally explain variance beyond active EMA in various outcomes (e.g., depression, anxiety, stress) (60).

Although it is possible to passively assess mood states or stress using a smartphone or a wearable device (61, 62), the measurement applications may not be validated compared to devices that track activity or sleep (e.g., accelerometry). Therefore, researchers who plan to include passive assessment of mood states need to be aware of this potential limitation. Currently, there are commercial (e.g., M-path or Movisens) and research platforms, some of which are open-source, available for passive sensing. A recently published guideline for e-health included recommendations for sensing research (63).

States of vulnerability were incorporated in the five JITAIs, but only one of five used passive EMA and complex machine learning techniques to measure and analyze states of vulnerability. Three systematic reviews demonstrated that when machine learning algorithms analyze passive data (e.g., call logs, social media use, or vocabulary of the text individuals write), future mental health states in both general and clinical populations could be predicted (61, 64, 65). The predictive accuracy of these models ranges from acceptable to very good (61, 64, 65). However, both reviews concluded that the field, despite advancing rapidly, is still in its early stages, with limited evidence supporting high-quality features for predicting mental states (61, 64, 65). In addition, the provision of adequate computational power and infrastructure required to accomplish the execution of machine-learning approaches in real-time remains a challenge. Furthermore, heterogeneity between studies is high, and assessment, modeling, and reporting procedures need to be critically reviewed (61, 66). This may explain why passive EMA and complex learning algorithms have been infrequently integrated into JITAIs for mental health until now. Finally, other complex analytic techniques including control systems engineering and Bayesian approaches, need to be developed to handle the enormous amount of dynamic data provided in JITAIs using sensors and smartphones (38, 61).

Another point of interest is that in three of the four JITAIs where decision points were specified, these decision points occurred after the user had completed an active EMA about the tailoring variable (49–52, 54–56). While active EMA can indicate vulnerability and receptivity to interventions at specific moments, its infrequent measurements throughout the day (typically only a few times) may miss critical moments (20, 21). This can compromise the “just-in-time” component of JITAI. Passive EMA analyzed on the fly using complex analytic techniques could reduce interval times between decision points, as seen in SenseSupport measuring glucose levels every five minutes (53), increasing the chance that interventions are delivered in-time. Nevertheless, there are potential therapeutic benefits of active EMA such as the empowerment of participants as they acquire knowledge about their personalized moods and activity dynamics. In turn, this knowledge may enable them to make informed behavioral changes, as well as improve communication with their therapist/health care provider. Based on EMA data, personalized feedback can be provided either by the therapist or via advanced algorithms.

Moreover, in the identified JITAIs, the proximal outcomes, tailoring variables, decision points, and decision rules were not adaptive over time and were uniform for all users (49–57). However, according to the conceptual framework, adaptivity across these core components is essential for JITAIs: this adaptivity ensures that the delivered interventions are tailored to meet the unique and changing needs of individuals, aiming to achieve the best possible outcome for each person (20, 21). Using complex machine learning algorithms, such as (deep) reinforcement learning enhanced adaptivity (19, 22, 23). This type of algorithm analyzes if an intervention prompts engagement and reduces the targeted proximal outcome (e.g., reduction in ruminative thoughts) (22, 23). By learning and adapting over time, these algorithms continuously improve the predictive power of the model (22, 23).

In conclusion, we recommend that JITAI developers in mental health adopt passive EMA more comprehensively. To effectively measure states of vulnerability and receptivity, developers should explore which features of passive data can serve as indicators for them. Utilizing passive EMA for measuring these states can also enhance the frequency of decision points, thereby facilitating timely intervention delivery. Passive and active EMA complement each other as well as more regular research instruments such as standardized retrospective questionnaires (60, 63). EMA enables more insightful gains in the personal dynamics of depressive symptoms as well as treatment outcome prediction. For EMA to work well, a substantial number of data points are required as well and sophisticated analytic methods are needed such as Bayesian models. In addition, complex analytic techniques such as machine learning or system identification could also be incorporated. Employing complex analytic techniques can further enable adaptivity of core components like decision points and rules across and within users. The validation of EMA and its analysis with machine learning models are still in development but making rapid improvements. Furthermore, there is still debate as to whether EMA measures different constructs than traditional measurements do. Recent publications provide recommendations for the incorporation of passive sensing and machine-learning based methods in health interventions (29, 63). Potential challenges with ethical, privacy, and security issues associated with mobile sensing should also be considered (29).

#### 4.1.2 The necessity of human involvement and patient subgroups

The developers of CBT+ argued that for BNSD patients, treatment-as-usual should accompany a JITAI due to the severity and complexity of BNSD symptoms (42). Similarly, a position paper on JITAIs for suicide prevention recommended professional guidance for severe symptoms, suggesting that smartphone interventions may be suitable for low-risk individuals but not for those at high risk of suicidal behavior (12). The meta-analysis encompassing 33 studies on the efficacy of JITAIs across various health-related areas found that combining complex analytic techniques including machine learning with human involvement in the JITAIs produced more significant effects than relying solely on machine learning algorithms (41). It should therefore be investigated whether, and if so for whom, human guidance in for example the decision rules and interventions should be incorporated. Due to the varied nature of many mental health disorders, JITAIs might work differently for different subgroups of patients. It would therefore be interesting to investigate differences in the effectiveness of JITAIs within subgroups based on patient profiles, aligning with the developers of CBT+ interventions (50, 52).

#### 4.1.3 The added value of JITAIs over existing interventions

Current studies show promising initial findings regarding usability, feasibility, and effectiveness (49, 51, 53–56). However, the number of studies conducted so far is limited and the conducted feasibility and (small-pilot) RCT studies do not allow robust meta-analytic conclusions (49, 51, 53–56). Additionally, longer follow-up periods and considerations for cost-effectiveness were lacking. Consequently, it is premature to draw definitive conclusions about the effectiveness of JITAIs and the added value of JITAIs over existing interventions in addressing mental health issues, a conclusion consistent with findings in other domains. For instance, a meta-analysis encompassing 33 studies across various health-related areas found moderate to large effect sizes for JITAIs compared to waitlist controls and non-JITAI interventions yet highlighted the general lack of statistical power in these studies (41). Similarly, reviews examining JITAIs for promoting physical activity (35) and reducing harmful substance use (38), consisting of 19 and 17 papers respectively, indicated that JITAIs were generally acceptable and showed some symptom reduction from pre- to post-intervention, but most studies lacked the statistical power to detect significant clinical effects.

None of the five included JITAIs used micro-randomized trials (MRT) in their development. It is advised to use innovative research methods such as the micro-randomized trial (MRT). An MRT is designed to investigate the effects of adaptive interventions in real-time and can complement the JITAI development (67, 68). This approach is not only recommended by Nahum-Shani et al. (20, 21), but is also recommended by the innovative Multiphase Optimization Strategy (MOST) framework (69). MOST is a novel framework developed to optimize interventions, including adaptive interventions. Another option is the use control optimization trials to optimize the individual and adaptive elements of interventions (70, 71). The advice is therefore that future studies, investigating the added value of JITAIs over existing interventions, be adequately powered and make use of innovative research methods.

#### 4.1.4 Different labels for similar interventions

In the field of smartphone intervention developments, there are also JITAI-like developments where developers label their interventions differently. This means that it is possible that interventions, when evaluated against the framework of Nahum-Shani et al. (20, 21), could be considered JITAI but are labeled differently, such as “Ecological Momentary Interventions” (EMI) (15, 22) or micro-interventions (72). The use of different labels leads to confusion and hinders collaboration between developers and the synthesis of evidence. Therefore, we recommend the consistent use of shared terms and conceptual frameworks, which is a specific challenge due to the multidisciplinary nature of JITAI developments.

### 4.2 Strengths and limitations

This review has several limitations. Firstly, we only included studies that self-identified as “JITAIs”. This may have resulted in missing (smartphone) interventions that could be considered JITAIs according to the Nahum-Shani et al.'s framework (20, 21). Secondly, all studies we found were conducted in the United States of America. This raises questions about the extent to which the results can be generalized to other countries. In addition, no quality assessments of the included studies were conducted due to the heterogeneity of the study designs. Nevertheless, the review also offers several strengths, including providing an overview of a new and relevant field for the prevention and treatment of mental health symptoms, a pre-registered study protocol, adherence to international reporting guidelines, and an extensive search in nine databases.

### 4.3 Conclusion

It is concluded that JITAIs for mental health domain are still in their early stages, with opportunities for improvement in both development and testing. For the future, we recommend that developers explore the use of passive EMA and complex analytical techniques including but not limited to machine learning to measure and analyze states of receptivity and vulnerability and to enhance in-time and adaptive delivery of the JITAIs. Additionally, evaluating the potential added value of human involvement and investigating patient subgroups, conducting fully powered studies using innovative research methods such as MRTs and the MOST framework, and adopting a consistent use of the term “JITAI” are also recommended. This will aid in optimizing personalization, effectiveness, and user engagement in JITAIs.

## Data Availability

The original contributions presented in the study are included in the article/Supplementary Material, further inquiries can be directed to the corresponding author.
