# Supplementary material for: Beyond the current state of just-in-time adaptive interventions in mental health: a qualitative systematic review
Source: Front Digit Health. 2025 Jan 28;7:1460167. doi: 10.3389/fdgth.2025.1460167 (PMC11811111; doi:10.3389/fdgth.2025.1460167)
Supplement: Supplementary file 1 [file Datasheet1.pdf]

Supplementary Material

1 Supplementary Table 1. Search Query's

Database	Search query
**PubMed**	"JITAI"[tiab] OR "JITAIs"[tiab] OR "just-in-time adaptive intervention*"[tiab] OR ("just-in-time"[tiab] AND adaptive[tiab]) OR "momentary intervention*"[tiab]
**Embase**	'jitai':ab,ti,kw OR 'jitais':ab,ti,kw OR 'just-in-time adaptive intervention*':ab,ti,kw OR (('just-in- time' NEAR/8 adaptive):ab,ti,kw) OR 'momentary intervention*':ab,ti,kw
**APA PsycInfo**	TI("JITAI" OR "JITAIs" OR "just-in-time adaptive intervention*" OR ("just-in-time" AND adaptive) OR "momentary intervention*") OR AB("JITAI" OR "JITAIs" OR "just- in-time adaptive intervention*" OR ("just-in-time" AND adaptive) OR "momentary intervention*") OR KW("JITAI" OR "JITAIs" OR "just-in-time adaptive intervention*" OR ("just-in-time" AND adaptive) OR "momentary intervention*")
**Cinahl**	TI("JITAI" OR "JITAIs" OR "just-in-time adaptive intervention*" OR ("just-in-time" AND adaptive) OR "momentary intervention*") OR AB("JITAI" OR "JITAIs" OR "just- in-time adaptive intervention*" OR ("just-in-time" AND adaptive) OR "momentary intervention*") OR SU("JITAI" OR "JITAIs" OR "just-in-time adaptive intervention*" OR ("just-in-time" AND adaptive) OR "momentary intervention*")
**Web of Science**	TS=("JITAI" OR "JITAIs" OR "just-in-time adaptive intervention*" OR "momentary intervention*") OR TS=("just-in-time" AND adaptive)
**Cochrane CENTRAL**	("JITAI" OR "JITAIs" OR "just-in-time adaptive intervention" OR "just-in-time adaptive interventions" OR ("just-in-time" AND adaptive) OR "momentary intervention" OR "momentary interventions"):ti,ab,kw
**ACM**	JITAI* OR "just-in-time adaptive intervention*" OR "momentary intervention*"
**DBLP**	JITAI|"just-in-time adaptive intervention"|"momentary intervention"
**Scopus**	TITLE-ABS-KEY("JITAI" OR "JITAIs" OR "just-in-time adaptive intervention*" OR "momentary intervention*") OR TITLE-ABS-KEY("just-in-time" AND adaptive)

2 Supplementary Material 1. JITAI development: Operationalization of JITAIs

Bulimia Nervosa Spectrum Disorders (BNSD): CBT+

Distal outcome. In CBT+, the distal outcome is skill enhancement, known from face-to-face CBT for BNSD to positively affect clinical outcomes (e.g., regular eating, binge eating management).

Proximal outcomes. CBT+ has two proximal outcomes, drawn from face-to-face CBT for BNSD. The first proximal outcome is defined as "skill practice," and the second is defined as "reaching

weekly goals" throughout the intervention. However, the skills and goals addressed can shift on a weekly basis, demonstrating the adaptability of the proximal outcome over time. Studies in face-to-face CBT settings showed that both proximal outcomes are associated with improved long-term skill enhancement (distal outcome) upon treatment completion.

Intervention options. The interventions, rooted in face-to-face CBT, provide psychoeducation and behavioral tips via text messaging to facilitate the achievement of proximal outcomes. The JITAI is delivered “in-time”. The decision on which CBT skill the CBT+ intervention should focus is “adaptive”; made based on the outcomes of the tailoring variables and decision rules, leading to varied interventions within and among individuals over the course of treatment.

Tailoring variables. CBT+ utilizes active EMA, such as self-reporting disordered eating behaviors as laxative use, as a tailoring variable. The JITAI considers vulnerability based on the EMAs to determine whether an intervention is warranted (e.g. whether a person reports laxative use), while state of receptivity is not considered. The choice of the tailoring variables was theoretically justified based on face-to-face CBT and are directly linked to proximal outcomes.

Decision points. In CBT+, the decision points occur immediately after participants complete active EMA. The JITAI adopts a personalized approach, requiring participants to self-initiate EMA completion, complemented by tailored reminders. The individually tailored reminder schedule is set by clinicians to match participants’ eating routines and can be adapted over time, as needed. No theory that supported the chosen decision points was explicated.

Decision rules. CBT+’s decision rules consist of a machine learning decision tree, that is established before JITAI initiation and are not adapted over time. No theory supporting the chosen decision points was explicated.

Bulimia Nervosa Spectrum Disorders: SenseSupport

Distal outcome. In SenseSupport, the distal outcome is long-term dietary restraint (i.e., decrease binge eating and establish a regulating eating pattern), corresponding to the clinical outcomes of face-to-face CBT for BSND.

Proximal outcomes. The proximal outcome is short-term dietary restraint. Practicing short-term dietary restraint during treatment is known to positively influence long-term dietary restraint.

Intervention options. SenseSupport provides CBT based supportive text-messages encouraging regular eating, contributing to reaching the proximal outcome. The interventions are delivered “in-time” and are “adapted” over time based on tailored variable outcomes, leading to varied interventions within and among individuals.

Tailoring variables. This JITAI uses passive monitoring of glucose levels with a sensor inserted just underneath the skin and a transmitter securely attached to a sensor, a method commonly utilized for managing diabetes patients. A machine learning algorithm analyses the sensor’s data to identify states of vulnerability, particularly focusing on disordered eating behaviors like purging. Disordered eating behaviors are directly associated with the proximal outcome, as empirically validated by face-to-face CBT practices. Receptivity is not considered as a tailoring variable.

Decision points. SenseSupport employs five-minute intervals between decision points for glucose monitoring sensor reading. No theory supporting the chosen decision points was explicated; the five-minute interval aligns with the standard interval of the sensor used.

Decision rules. The JITAI utilizes a parameter invariant-algorithm to analyze real-time glucose monitoring data for the detection of disordered eating behavior (tailoring variable). The rules are predetermined and not adapted throughout the intervention period for all participants. The choice of the decision rules was not theoretically justified.

Depression: JITAI-MRFCBT

Distal outcome. The distal outcome is a clinical outcome, namely reducing depressive rumination.

Proximal outcomes. JITAI-MRFCBT's proximal outcome is the disruption of ruminative thoughts. This is formulated based on established evidence from face-to-face rumination focused CBT and serves as a short-term goal hypothesized to influence the distal outcome. This proximal outcome is not adapted throughout the intervention duration.

Intervention options. The interventions are messages and training exercises, with content derived from face-to-face RF-CBT and known to influence the proximal outcome. The interventions are delivered “in time” and “adaptive”. The decision which message or exercise is send is based on the outcomes of the tailoring variables and decision rules, leading to varied interventions within and among participants over the course of treatment.

Tailoring variables. The tailoring variables consist of a combination of multiple active EMAs. The active EMAs focuses on vulnerability by asking whether stressful events occurred in the past three hours, and if so, whether they led to rumination, along with questions about the nature of the stressor. Additionally, the daily questionnaire assesses receptivity by asking participants about their engagement in other activities that might influence receptivity for the intervention. The choice of each tailoring variables was theoretically justified based on face-to-face RF-CBT.

Decision points. Decision points occur directly after the active EMA. Identical decision points are adopted for each participant and remain stable throughout the intervention period. No theory supporting the chosen decision points was explicated.

Decision rules. JITAI-MRFCBT's decision rules consist of a decision tree, that is established before JITAI initiation and not adapted over time. The choice of the decision rules was not theoretically justified.

Insomnia: iREST

Distal outcome. The distal outcome is the clinical outcome, namely reduced insomnia, corresponding to face-to-face CBT for insomnia.

Proximal outcomes. Not specified.

Intervention options. The interventions are sleep tips delivered through text-messaging. iREST interventions are not delivered "in-time" as they involve clinician decision-making before delivery (tailoring variable). However, the interventions are adaptive in that the sleep tips personalized over time based on tailored variable outcomes, resulting in varied interventions within and among

individuals. The sleep tips were informed by empirical evidence face-to-face CBT for insomnia interventions that supported positive impacts from these tips on the distal outcome.

Tailoring variables. iREST uses a combination of multiple tailoring variables, measured through various modalities. These include active EMA (i.e., wake and sleep logs), weekly self-reported outcome measures (e.g., PTSD), and clinician decision-making. State of vulnerability and receptivity are not considered.

Decision points. The decision points occur immediately after participants completed active EMA and were identical for each participant throughout the entire intervention period. However, before an intervention is send, the clinician must then give a "go" or "no-go". The rationale behind the chosen decision points was not explicitly stated by theory.

Decision rules. The initial step in iREST's decision rules involves a machine learning decision tree, where the algorithm recommends an intervention based on active EMAs and weekly self-reported measures. Subsequently, the participant's clinician makes the final decision before administering the intervention. No theory supporting the chosen decision rules was explicated.

Maternal prenatal stress: Wellness-4-2

Distal outcome. The distal outcome is a clinical outcome, namely improving foetal neurodevelopment (i.e., fetal brain development).

Proximal outcomes. The proximal outcome of Wellnes-4-2 is reducing maternal prenatal stress. This short-term goal is derived from CBT-based interventions and hypothesized to influence the distal outcome of improving fetal neurodevelopment. The proximal outcome is stable throughout the intervention duration.

Intervention options. The interventions are CBT-based text messages focusing on skill reinforcement and mindfulness. Empirical evidence supports the positive impacts of the intervention on the proximal outcome maternal prenatal stress. The interventions are delivered “in-time” and “adaptive”. The decision which text message is send is based on the outcomes of the tailoring variables and decision rules, leading to varied interventions within and among participants over the course of treatment.

Tailoring variables. Wellness-4-2 integrates active EMA of self-reported stress with passive EMA via unobtrusive ECG sensors to measure heart rate variability, along with the content of the most recent face-to-face stress-reduction course session. The state of vulnerability (elevated stress levels) is determined based on both active and passive EMA data. Receptivity is not taken into consideration as tailoring variable.

Decision points. Not specified.

Decision rules. Wellness-4-2’s decision rules consist of a decision tree, that is established before JITAI initiation and not adapted over time. No theory supporting the chosen decision points was explicated.

3 Supplementary Material 2. Narrative descriptions of the conducted studies

Bulimia Nervosa Spectrum Disorders: CBT+

Feasibility study. The first paper detailed the development of CBT+ and presented findings from a small proof-of-concept pilot trial that involved five BNSD patients and three therapists (49). Patients who received 16 weekly CBT sessions alongside CBT+ found the JITAI a relatively low burden. Though adherence to self-monitoring in the application was high. Both patients and clinicians found CBT+ useful; however, a 40% treatment dropout rate (two out of five patients) has prompted concerns about feasibility.

Randomized controlled trial (RCT). The next paper investigated the feasibility, acceptability, engagement, and treatment outcomes of CBT+ in an RCT ($n = 55$) (51). Patients receiving 16 weekly CBT sessions were randomized to receive CBT+ JITAI (experimental group; $n = 29$) or no JITAI (control group; $n = 26$). According to the patients in the experimental group, CBT+ demonstrated to be feasible and acceptable. By CBT session 16, BNSD-skill use had increased, and bulimia nervosa symptoms were significantly decreased in both groups (within-group p's < 0.05). No significant differences were found between the experimental and the control groups across treatment outcomes (between-group all p's > 0.05)

Secondary analysis. A secondary analysis used data from the above mentioned RCT ($n = 55$) (51) to explore whether baseline emotional regulation and impulsiveness moderated treatment outcomes (50). Findings showed that in the group receiving CBT+ (experimental group; $n = 29$), greater baseline emotional regulation and impulsivity were associated with better treatment outcomes (within-group p's <0.05). Conversely, in the non-JITAI group (control group; $n = 26$), lower emotional regulation difficulties and impulsivity were linked to better treatment outcomes (within-group p's <0.05).

Full factorial randomized controlled trial. This paper outlined a 2x3 full factorial RCT protocol (aiming for $n = 264$) (52). It builds on the secondary analysis of the previous RCT (51) to investigate whether baseline emotional regulation deficits influence the optimal intervention intensity level. The trial aims to determine the best intervention intensity for two common mobile intervention components—self-monitoring and micro interventions—combined with 16 weekly CBT sessions. Patients were randomized to one of the six groups, determined by a combination of the different options of self-monitoring and micro-interventions. Self-monitoring options included (1) standard self-monitoring plus monitoring of BNSD treatment skills, and (2) standard self-monitoring of eating behaviors, emotions, and thoughts. Micro intervention options comprised (1) the CBT+ JITAI, (2) automated reminder messages prompting patients to practice BNSD treatment skills at semi-random intervals, and (3) no micro intervention. Potential findings could support tailoring the intensity of future interventions based on individuals' emotional self-regulation profiles.

Bulimia Nervosa Spectrum Disorders: SenseSupport

ABAB design study. This paper investigated SenseSupport's feasibility, acceptability, and initial (treatment) outcomes ($n = 30$), alongside 12 weekly face-to-face CBT sessions (53). Employing an ABAB design, patients acted as their own control, alternating between two-week periods without JITAI ("A") and two-week periods with JITAI ("B") during treatment. Results showed that patients found SenseSupport acceptable, but sensor retention was low due to connectivity issues. During JITAI periods ("B"), patients were less intent to maintain dietary restrictions (between-effect p <

0.05), but no other significant differences in eating behaviors were found (between-effects p's > 0.05). Pre-to-post treatment, significant decreases in binge eating, compensatory behaviors, and overall pathology were observed (within-group, p's < 0.05).

Depression: JITAI-MRFCBT

Pilot randomized controlled trial. This paper reported the findings of a pilot RCT testing JITAI-MRFCBT in persons with major depressive disorder ($n = 18$), recruited from the general population (54). Participants were randomly assigned to either receive JITAI-MRFCBT (experimental group; $n = 9$) or no treatment (control group; $n = 9$). Both groups showed a significant decrease in rumination episodes and time spent ruminating (within-group p's < 0.05). However, the decrease was significantly greater in the experimental group compared to the control group (between-group p's < 0.05).

Insomnia: iREST

Development and pilot usability. This paper described the development of iREST and showed results of the pilot study on the usability of iREST in active-duty service members and veterans aged 18-60 ($n = 19$) (55). iREST was perceived as highly usable; self-monitoring was high, and patients were satisfied with the application and would consider using it in the future.

Feasibility. The subsequent paper evaluated the pre-to-post intervention change in insomnia severity using iREST in a sample of military service members and veterans (experimental group, $n = 27$) (56). The efficacy of iREST was compared with face-to-face evidence-based insomnia CBT by extracting data from previously published trials (control group). The results demonstrated that the experimental group experienced significant improvements in insomnia, sleep quality, and nocturnal behavior from pre- to post-intervention (within-group p's < 0.05), with treatment outcomes that were non-inferior to the control group (between-group p's > 0.05).

Maternal prenatal stress: Wellness-4-2

Protocol paper. This paper discussed opportunities and challenges in tailoring and utilizing active and passive monitoring interventions for maternal prenatal stress (57). It highlighted the potential of JITAIs for maternal prenatal stress, since behavioral and physiologic responses to stress vary extensively per individual and over time. The authors described the protocol of an RCT ($n = 100$), with the experimental group receiving a 12-week prenatal stress reduction course and the Wellness-4-2 JITAI (anticipated $n = 50$), while the control group only monitor their stress using active EMA (anticipated $n = 50$).
